# Temporomandibular joint disorders as the only manifestation of juvenile idiopathic arthritis: a case report

**DOI:** 10.1590/S1679-45082018RC4003

**Published:** 2018-09-09

**Authors:** José Renato Ribeiro Pinto, Irineu Gregnanin Pedron, Estevam Rubens Utumi, Milton Edson Miranda, Elisa Cruz Pereira Pinto, Leopoldo Penteado Nucci

**Affiliations:** 1 Hospital Israelita Albert Einstein, São Paulo, SP, Brazil.; 2 Universidade de São Paulo, São Paulo, SP, Brazil.; 3 Hospital de Aeronáutica de São Paulo, SP, Brazil.; 4 Faculdade de Medicina e Odontologia São Leopoldo Mandic, Campinas, SP, Brazil.; 5 Centro Universitário do Planalto Central, Brasília, DF, Brazil.

**Keywords:** Arthritis, juvenile, Temporomandibular joint disorders, Mandibular condyle, Artrite juvenil, Transtornos da articulação temporomandibular, Côndilo mandibular

## Abstract

Juvenile idiopathic arthritis is a term used to include all chronic childhood arthritis of unknown etiology. It is characterized by chronic inflammation persisting for at least 6 weeks, beginning before 16 years of age. The characteristics present are chronic synovitis, arthralgia, impaired joint mobility in at least one joint, and erosion with destruction of cartilage and subchondral bone, that could be associated or not with systemic involvement, according to each subtype of the disease. During the pathologic process, the temporomandibular joint can be involved by the juvenile idiopathic arthritis, resulting in severe mandibular dysfunction, with higher frequency in female patients. Initially, these lesions can show minor alterations like flattening of the condyle, erosions, and evolve to severe lesions, like destruction of the head of the condyle. We report a case of male patient who had destruction of both condyles, as a result from juvenile idiopathic arthritis. Proposed mechanisms to explain the juvenile idiopathic arthritis was reviewed. In this report the patient did not have pain or inflammatory process, and the temporomandibular diseases was the only manifestation.

## RESUMO

Artrite idiopática juvenil é um termo usado para incluir toda artrite infantil crônica de etiologia desconhecida. É caracterizada por uma inflamação crônica, que persiste por pelo menos 6 semanas, com início antes dos 16 anos de idade. As características presentes são sinovite crônica, artralgia, mobilidade articular diminuída em pelo menos uma articulação, e erosão com destruição da cartilagem e do osso subcondral, podendo ser associada ou não com o envolvimento sistêmico, de acordo com cada subtipo da doença. Durante o processo patológico, a articulação temporomandibular pode ser envolvida pela artrite idiopática juvenil, resultando em disfunção mandibular severa, com maior frequência em pacientes do sexo feminino. Inicialmente, estas lesões podem mostrar pequenas alterações, como achatamento do côndilo e erosões, e evoluir para lesões graves, como a destruição da cabeça do côndilo. Relatou-se o caso de um paciente do sexo masculino, que apresentou destruição de ambos os côndilos, como resultado da artrite idiopática juvenil. Os mecanismos para explicar a artrite idiopática juvenil foram revisados na literatura. Neste relato de caso, o paciente não apresentou dor e nem processo inflamatório, sendo o comprometimento da articulação temporomandibular a única manifestação.

## INTRODUCTION

Juvenile idiopathic arthritis (JIA) is a term used to include all chronic childhood arthritis of unknown etiology (term established by the International League of Associations for Rheumatology). It is characterized by chronic inflammation that lasts for at least 6 weeks, beginning before age 16 accordingly to the criteria of the American College of Rheumatology.^(^ ^1^ ^,^ ^2^ ^)^

The temporomandibular joint (TMJ) can be damaged by JIA. Temporomandibular diseases (TMD) such as clicking sound, crackle, and disc disarticulation in patients with JIA could be responsible for drastic modifications as hypoplastic condyles, convex facial morphology, and retrognathia.^(^ ^1^ ^,^ ^2^ ^)^

It is a disease that occurs worldwide in all races and ethnic groups; however, the lack of an universal classification makes difficult to determine its incidence and prevalence rates, producing, therefore, conflicting data.^(^ ^2^ ^-^ ^5^ ^)^

A new denomination of JIA replaced others and the terms “chronic juvenile arthritis” and “juvenile rheumatoid arthritis” are used pursuant to geographic area. Juvenile idiopathic arthritis, based on the new denomination criteria, is redivided based on the onset of clinical aspects: oligoarthritis, arthritis disturbing one or at least four joints during the first 6 months of the illness; polyarthritis, more than four joints during the first six months; and the systemic form, arthritis correlated with marked systemic symptoms. Other categories are psoriatic arthritis and enthesitis related arthritis.^(^ ^2^ ^)^

The etiology is still unclear, but infections, physical traumas, genetic susceptibility, immunologic disturbances and stress have been mentioned as important factors.^(^ ^3^ ^,^ ^6^ ^)^ Juvenile idiopathic arthritis pathogenesis is still low versed: the interrelation between environmental factors and many genes has been considered as the most relevant working instrument to the enlargement of JIA. The idea that a number of microbes that colonize or infect not only the mucosal surfaces, like the oral cavity, but also the airways and gut might precipitate autoimmune processes, culminate in chronic arthritides, and JIA was first delineated at the beginning of last century.^(^ ^6^ ^)^

During the deleterious process, the TMJ, like any other joint, can be affected by the JIA, culminating in austere mandibular debilitation including reduced chewing ability, malocclusion and micrognathia.^(^ ^7^ ^)^ Modifications in facial anatomy can be an effect of growth disorder, associated with condylar damages.^(^ ^8^ ^,^ ^9^ ^)^ During the evolution, these lesions show low alterations, flattening of the condyle, erosions, or severe damages, like ruination of the head of the condyle.^(^ ^3^ ^)^

The diagnosis is clinical, and a few patients show atypical signs and symptoms at the initiation, delaying the diagnosis and treatment of the illness. The radiographic images are helpful for the diagnosis of the JIAs, allowing earlier detection of disease and, therefore, early intervention. The TMD may be first or even the only sign of the JIA.^(^ ^3^ ^,^ ^10^ ^,^ ^11^ ^)^

## CASE REPORT

**Figure 1:**
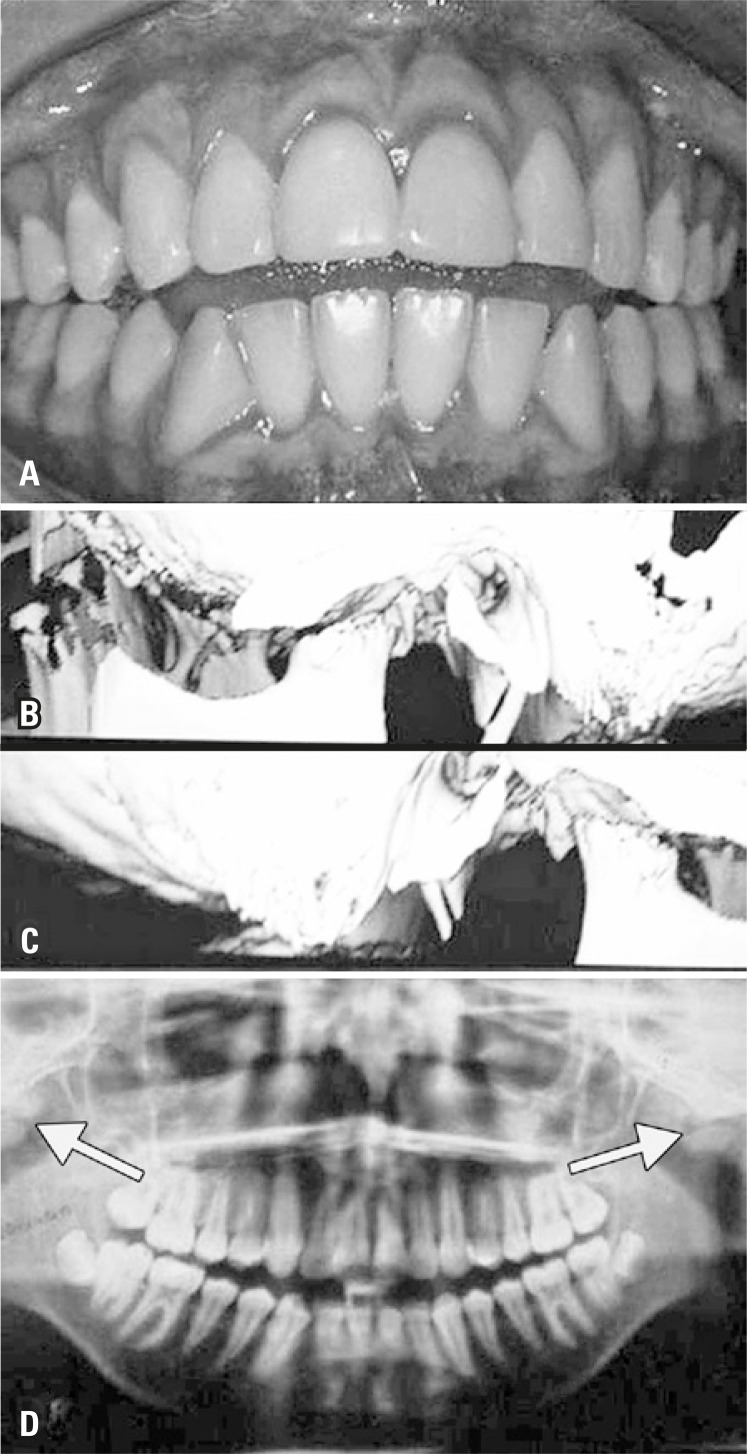
Acute changes in the temporomandibular joints of patient with juvenile idiopathic arthritis, as detected by 3D-computed tomography and panoramic X-ray. The clinical photo of patient show anterior open bite (A). Chronic changes in the temporomandibular joints are shown on sagittal images by three-dimensional computed tomography (B and C), involving condylar flattening, condylar erosions and anterior disk displacement. The same observations were show by panoramic X-ray (D)

A male Caucasian patient, 12 years old, was referred by his orthodontist for evaluation of his TMJ, since the panoramic radiography showed bilateral flat condyles. Clinically, the patient showed micrognathia, retrognathia and anterior open bite (Figures 1A and D). Functionally, mouth opening, speaking and chewing were normal. During opening, closing and lateral excursion, no sounds, discomfort or symptoms were present. Since the patient did not complain of any symptoms or difficulty in lateral excursions, a three-dimensional computed tomography was requested. He was referred to a rheumatologist who requested several laboratory tests. Of these, the only positive result was for antinuclear antibody (ANA): 1:80 (reference value – 1:40), by the indirect imunofluorescence method.

The clinical diagnosis was inconclusive for JIA, systemic lupus erithematosus, or any other disease. However, the 3D-computed tomography findings showed major erosion of the head of both condyles (Figures 1B and C). Facial morphological alteration, retrognathia, micrognathia and condyle erosion, even without inflammatory signs and no pain, led to a JIA diagnoses. No invasive treatment was performed but the patient underwent annual follow-up.

After 2 years, the patient was examined and no clinical symptoms were observed. At this time, a new three-dimensional computerized radiography was requested. The erosion process seemed to be stabilized. The patient had no difficulties in lateral excursions, and no pain or signs of inflammatory process. The only difference was in the ANA laboratory test, showing positive result: 1:60 (reference value: 1:40), by the indirect imunofluorescence method.

Since the patient had no alterations in his radiographic or clinical status, no drugs were prescribed. Although the ANA reduced in the last 2 years, we asked the patient to be examined again by a rheumatologist. The diagnosis was inconclusive one more time.

Between the first appointment and the last clinical examination, magnetic resonance imaging was performed to evaluate bone and soft tissues changes, and there were no morphological modifications of the condylar region, and no modifications of the patient profile and anterior open bite. Since the aim of the treatment of patients with JIA is the control of the pain and inflammatory activity, no drugs were prescribed.

After 11 years since the first consultation, no signs of evolution of the disease was observed and the patient did annual check-ups (clinical and radiographic examination).

## DISCUSSION

In JIA, the involvement of the masticatory system, especially involving the TMJ by chronic inflammatory process, is relatively common. Mandibular alterations in size and form can occur, once the pathological changes affect the condyle, center of mandibular growth. Children affected by JIA present a rate of 28.9% of condylar abnormalities, 40% of them being asymmetrical. Some authors present TMD as initials manifestations of the JIA or the only manifestations.^(^ ^3^ ^,^ ^9^ ^)^

Scientific articles reports a 12%-predominance of TMD in juvenile patients classified by image exams. These results denote that rheumatic illness can conduct to a higher number of TMD than in the normal community. It is arduous to compare the clinical results of our TMJ investigation with those of the scientific literature because there is no classification for juvenile patients, such as the clinical diagnostic criteria for TMD classification, related with adult patients.^(^ ^3^ ^)^ A literature review reveals that clicking, crackle, and pain are the most found criterions for the study of the juvenile TMJ. Moreover, the clinical symptoms of juvenile TMD change during the growth of the patients, making the analysis difficult, because previous published scientific articles did not divide patients into different age groups.^(^ ^2^ ^,^ ^7^ ^,^ ^11^ ^)^

The clinical appraisal of the TMJ is arduous, mainly in small children, by reason of symptoms such as pain could often be checked only circumlocutory by asking the parents of patients.^(^ ^5^ ^)^ Additionally, clinical symptoms could be camouflaged by antirheumatic treatment. Prior therapy of TMD could avoid austere malfunctions of the TMJ caused by hypoplastic condyles and growth modifications of the mandible.^(^ ^7^ ^,^ ^8^ ^)^ The most important aspect of the related case is the severe TMD as the only manifestations of JIA in a male patient, with 12 years old that was followed up by 11 years.

Radiographic images have been used for the diagnosis of JIA. However the difficulty to determine an adequate therapeutic program has been related to the low sensitivity of standard radiographic techniques. To evaluate the clinical symptoms, a good clinical history of the patient is mandatory. The TMJ radiography alone is not enough. Plain radiographs are difficult to interpret due to bone overlap; computed tomography provides bone details. However it exposure patients to significant radiation. Magnetic resonance is the gold standard method to demonstrate bone and soft tissues pathologies.^(^ ^6^ ^)^ Magnetic resonance imaging and ultrasonography have no risk of radiation and are advantageous over computed tomography.^(^ ^9^ ^-^ ^11^ ^)^

Temporomandibular diseases are often camouflaged by antirheumatic treatment. From the dentist’s perspective, a restorative intervention, splint treatment, should initiate during the first stages of the TMD.^(^ ^6^ ^,^ ^7^ ^)^

Another studies with numerous patients from distinct age groups are needed to obtain more details about TMJ damages in patients with JIA. This would also allow statistical interrelations between the ultrasonography and clinical results. The significant interrelations between pathological image findings, the perpetuation of the rheumatic illness, and the number of altered peripheral joints make the approach attractive for use as a screening technique.

## CONCLUSION

During the pathologic process, the temporomandibular joint can be involved by the juvenile idiopathic arthritis, resulting in severe mandibular dysfunction, with higher frequency in female. The temporomandibular diseases may be first or even the only sign of the juvenile idiopathic arthritis. When the patients do not report pain or inflammatory process, a non-invasive treatment should be performed including annual consultations and radiographic examination.
